# Towards decentralization of *Salmonella* serotyping and risk assessment in poultry production environments with nanopore sequencing

**DOI:** 10.3389/fmicb.2025.1669089

**Published:** 2025-10-15

**Authors:** Ruwani Karunarathna, Chao Chun Liu, Dhinesh Periyasamy, Arnold Berg, Musangu Ngeleka, Anatoliy Trokhymchuk

**Affiliations:** ^1^Department of Veterinary Pathology, Western College of Veterinary Medicine, University of Saskatchewan, Saskatoon, SK, Canada; ^2^Prairie Diagnostic Services, Saskatoon, SK, Canada; ^3^Department of Molecular Biology and Biochemistry, Simon Fraser University, Burnaby, BC, Canada; ^4^Department of Large Animal Clinical Sciences, Western College of Veterinary Medicine, University of Saskatchewan, Saskatoon, SK, Canada

**Keywords:** *Salmonella* serovars, poultry, nanopore whole genome sequencing, antimicrobial resistance, virulence

## Abstract

*Salmonella enterica* is a highly diverse group of organisms with over 2,500 serovars described to date. However, not all serovars are pathogenic to humans and animals. Many serovars typically cause self-limiting gastrointestinal infections, but certain host-adapted serovars, as well as those expressing specialized virulence factors (e.g., Vi capsule) have an increased potential to cause bacteremia and trigger systemic responses, necessitating immediate medical attention. Timely identification of *Salmonella* at the serovar level is, therefore, critical for informing risk assessments, guiding clinical decision-making and implementing effective strategies to prevent and control both environmental contamination and clinical infections. Yet, access to *Salmonella* serotyping services remains limited and unevenly distributed worldwide, causing substantial delays in responses to contamination and outbreaks, particularly in remote communities and under-resourced settings, Nanopore sequencing has emerged as a highly portable platform with a relatively low cost of entry that renders it an ideal technology to enable the widespread decentralization of *Salmonella* serotyping by sequencing. Here, we introduce a robust and accurate end-to-end laboratory workflow that utilizes long-read nanopore sequencing to generate *Salmonella* whole genome sequencing (WGS) data at a quality and depth sufficient for *Salmonella* risk assessment in routine diagnostic settings We validated our workflow against a panel of 16 serovars comprising 80 environmental isolates sampled from Canadian poultry farms, yielding 100% accuracy in serovar prediction. We showed that our workflow can obtain serotyping results and identify genetic risk factors within 24 h of bacterial isolation—a significant improvement over the typical 3 weeks turnaround in Canada. We additionally demonstrated the added benefits of WGS by annotating the draft genome assemblies generated from our laboratory workflow to identify virulence factors and antimicrobial resistance (AMR) determinants, leading to our discovery of previously uncharacterized genetic signatures putatively associated with *Salmonella* adaptation to avian environments, such as aerobactin biosynthesis genes. The genomic analyses presented herein have been packaged as an open-source, user-friendly sequence analysis pipeline that facilitates standardized, reproducible, scalable and portable analysis of *Salmonella* surface antigen markers and genetic risk factors associated with pathogenicity and AMR. Our protocol provides a comprehensive toolkit that empowers regional and local laboratories to independently conduct *Salmonella* risk assessments at scale—an essential step toward establishing real-time genomic surveillance and equitable access to contemporary diagnostic technologies.

## Introduction

Non-typhoidal salmonellosis (NTS) accounts for significant public health burden, ranging from self-limiting gastroenteritis to life threatening systemic infections (Popa and Papa, 2021). More than one million NTS cases are reported annually in the United States alone. The predominant source of these infections is from the consumption of contaminated animal-derived products (CDC, n.d.). Domestic and wild animals, such as layer chickens and wild birds act as asymptomatic carriers of *Salmonella* and shed the organism in food production environments leading to table eggs and carcass contamination. In addition to the inconspicuous nature of the disease in animal hosts, the problem is further compounded by the persistence of the pathogen at multiple points along the farm-to-fork continuum (e.g., animal feeds, food processing equipment, conveyers) leading to continuous propagation and cross-contamination for extended periods of time. Identifying risk factors contributing to health burdens caused by enteric pathogens including NTS requires active monitoring of human illnesses and contamination of water, farm and retail sources.

Central to *Salmonella* surveillance is serotyping, a method for differentiating *Salmonella* strains based on the agglutination of antisera sensitive to molecular variations in the somatic (O) and flagellar (H) antigens present on bacterial cell surfaces. This method divides the enterica species into over 2,500 serovars, of which only less than 200 are considered pathogenic to humans (Popoff et al., 2004). Performing serotyping *Salmonella*-positive cultures is, therefore, critical for assessing the pathogenic potential of the isolated strain and the degree of risk posed to public and animal health. In addition, serotyping plays an important role in source attribution during epidemiological investigations of foodborne outbreaks, as the serovar identity of the outbreak strain could be indicative of the sources of contamination (Diep et al., 2019). *Salmonella* source attribution is in part driven by the intra-host evolution of the pathogen which has manifested significant diversity in host range across different serovars. The loss of adhesins or fimbriae critical to host tissue attachment and the acquisition of novel virulence factors to reduce local competition have been implicated in giving rise to the remarkable range of specialized serovars adapted to different environments. Common examples include specialization in humans (*S. typhi*), swine (*S. choleraesuis*), bovine (*S*. Dublin) and birds (*S*. Kentucky, *S. gallinarum*) (Uzzau et al., 2001).

However, the current turnaround time from sample collection to serovar identification is inadequate. In Canada, it takes on average, three to 4 weeks to obtain *Salmonella* serotyping results, leading to considerable delays in reporting and decision-making. As of this writing, the Public Health Agency of Canada (PHAC) is the primary provider of this service with substantial government subsidies to keep the service fee affordable and competitive (Government of Canada, 2024). Clients requesting *Salmonella* serotyping are expected to send pure bacterial isolates to the Office International des Epizooties (OIE) Reference Laboratory for Salmonellosis in Guelph, Ontario, where tests are performed. The centralization of this service in Eastern Canada imposes an inherent delay due to sample transportation, costing up to 48 h depending on the shipment’s origin (e.g., Central Canada and Western Canada). Furthermore, samples are not guaranteed to be processed immediately upon arrival. In our experience, the time to reporting excluding transportation time could range from 2 to 3 weeks with priorities given to samples suspected to be high risk serovars (e.g., *Salmonella Enteritidis*). While regional health laboratories in Canada may provide quicker turnaround times for clients within their respective regions, most of these laboratories, to our knowledge, have a very limited detection range, typically targeting only a small number of serovars.

Delayed notification of the active circulation of high-risk clones in the food supply chain can have severe consequences for both producers and consumers. For instance, commercial egg producers may unknowingly continue supplying retailers while their layer flocks are infected with pathogenic serovars, thereby exposing consumers to significant health risks. NTS outbreaks linked to food consumption remain a persistent public health challenge, with occasional fatalities reported, highlighting both the burden NTS places on healthcare systems and the urgent need for earlier detection and reporting of high-risk clones along the farm-to-fork continuum. In recent decades, whole genome sequencing (WGS) has emerged as a rapid and cost-effective means to profile microbial organisms, leading to its broad adoption by hospitals, national surveillance networks and government health agencies. While WGS offers great promises to improve clinical management and pathogen surveillance, transitioning from conventional phenotypic assays to genomic approaches remains highly challenging for many regional and under-resourced laboratories. Key barriers include: (1) the substantial capital investment required to acquire sequencing instruments, (2) a shortage of trained personnel capable of conducting sequencing, and (3) limited informatics expertise and computational infrastructure to process and analyze high-throughput sequencing data.

To address these gaps, we developed and validated an end-to-end laboratory workflow using Oxford Nanopore Technologies (ONT) nanopore sequencing (NS) to generate *Salmonella* WGS data at a quality and depth sufficient to inform risk assessments in food production settings. ONT sequencing devices are increasingly popular among laboratories with small to medium-sized budgets, as they can be acquired at a substantially lower upfront cost compared to other platforms such as Illumina or PacBio. While NS has been evaluated for *Salmonella* serotyping in prior studies, the technology has not yet been systematically assessed in a Canadian context. Here, we demonstrate that NS enables *Salmonella* serotyping within 24 h of bacterial isolation, while producing results comparable to those generated by the current Canadian gold standard—Illumina sequencing, as practiced by the OIE Reference Laboratory for Salmonellosis. To complement our laboratory workflow, we developed an open-source bioinformatics pipeline, *SamnSero*, which consolidates the necessary computational tools to process NS data and generate actionable reports. Together, our laboratory and computational workflows equip laboratories with the tools and knowledge needed to independently perform sequencing-based *Salmonella* diagnostics—a critical gap that continues to hinder sequencing adoption (Davedow et al., 2022). By lowering technical and financial barriers, our work aims to catalyze the expansion of national and international sequencing capacity, enabling *Salmonella* diagnostics and surveillance to be conducted at a scale and speed that meet both public health and industry needs.

## Results

### *Salmonella* serovar prediction and genome quality assessment

**Figure 1:**
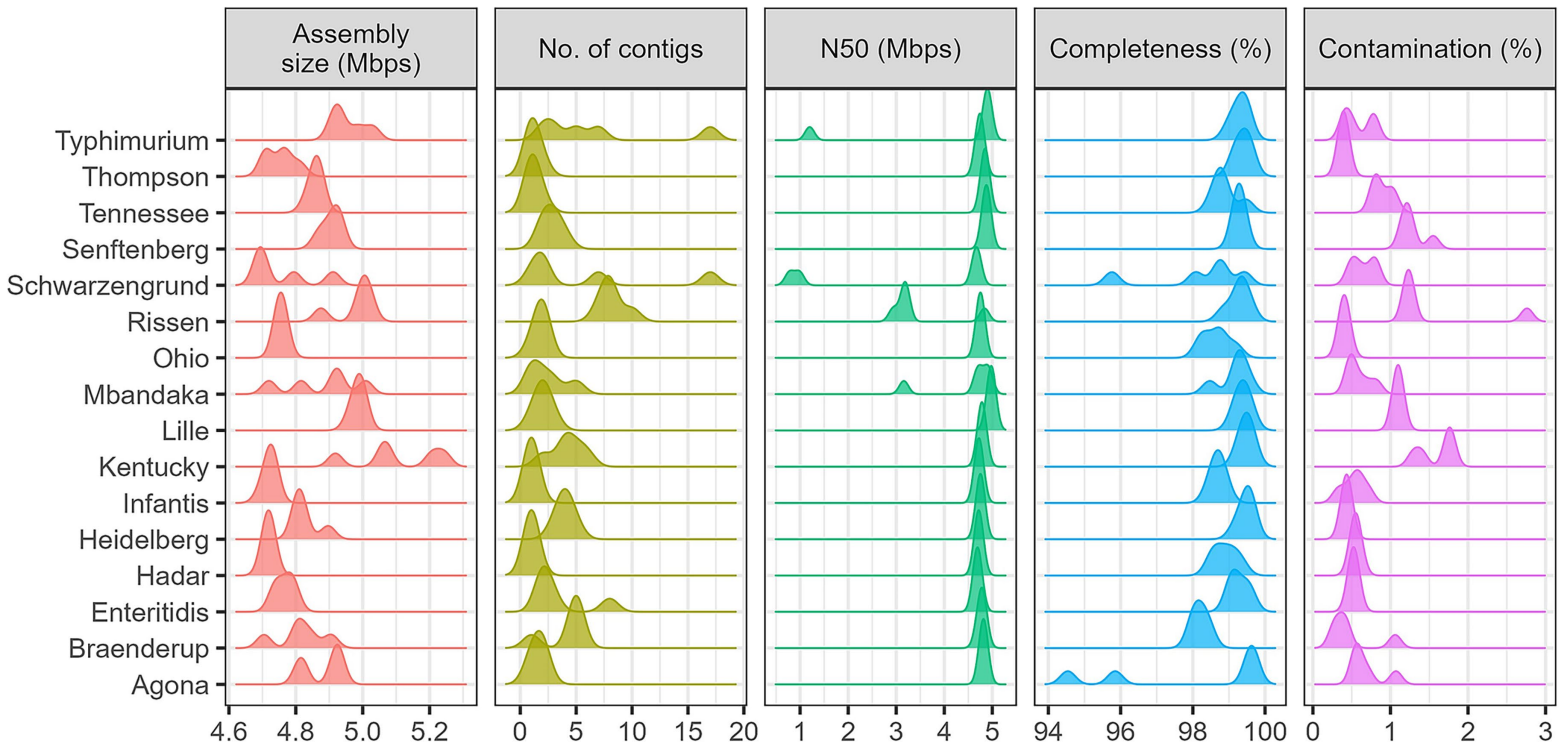
Summary of genome assembly quality metrics across the 80 *Salmonella* isolates. The average assembly size of *Salmonella* genome is 4.85 Mbps (95% CI: ±0.0265) which is falling within the expected range of *Salmonella* genome sizes. The average N50 was 4.54 Mbps (95% CI: ±0.1794) and 86% (69/80) of the assemblies carried fewer than five contigs while Majority of assemblies obtained 1 or 2 total contigs. 96% (77/80) of the assemblies carried >98% of single-copy *Salmonella enterica* species marker genes (indicator of genome completeness), less than 1% of which were found duplicated (indicator of genome contamination). The average N50 was 4.54 Mbps (95% CI: ±0.1794) and 86% (69/80) of the assemblies carried fewer than five contigs.

We observed 100% concordance in *Salmonella* serovar identification between our data, obtained by nanopore WGS and the Illumina short-read WGS results reported by PHAC (Supplementary Table S1). We attributed the high accuracy of *in silico* serotyping to the robust quality of the nanopore sequencing data and the resulting assemblies. The nanopore genome assemblies yielded an average assembly size of 4.85 Mbps (95% CI: ±0.0265; Range: 4.68–5.24 Mbps), falling within the expected range of *Salmonella* genome sizes. 96% (77/80) of the assemblies carried >98% of single-copy *Salmonella enterica* species marker genes (indicator of genome completeness), less than 1% of which were found duplicated (indicator of genome contamination). The nanopore assemblies were also highly contiguous. The average N50 was 4.54 Mbps (95% CI: ±0.1794; Range: 0.78–4.98 Mbps) and 86% (69/80) of the assemblies carried fewer than five contigs (Supplementary Table S1 and Figure 1).

### *In vitro* and in silico assessments of antimicrobial resistance (AMR)

**Figure 2:**
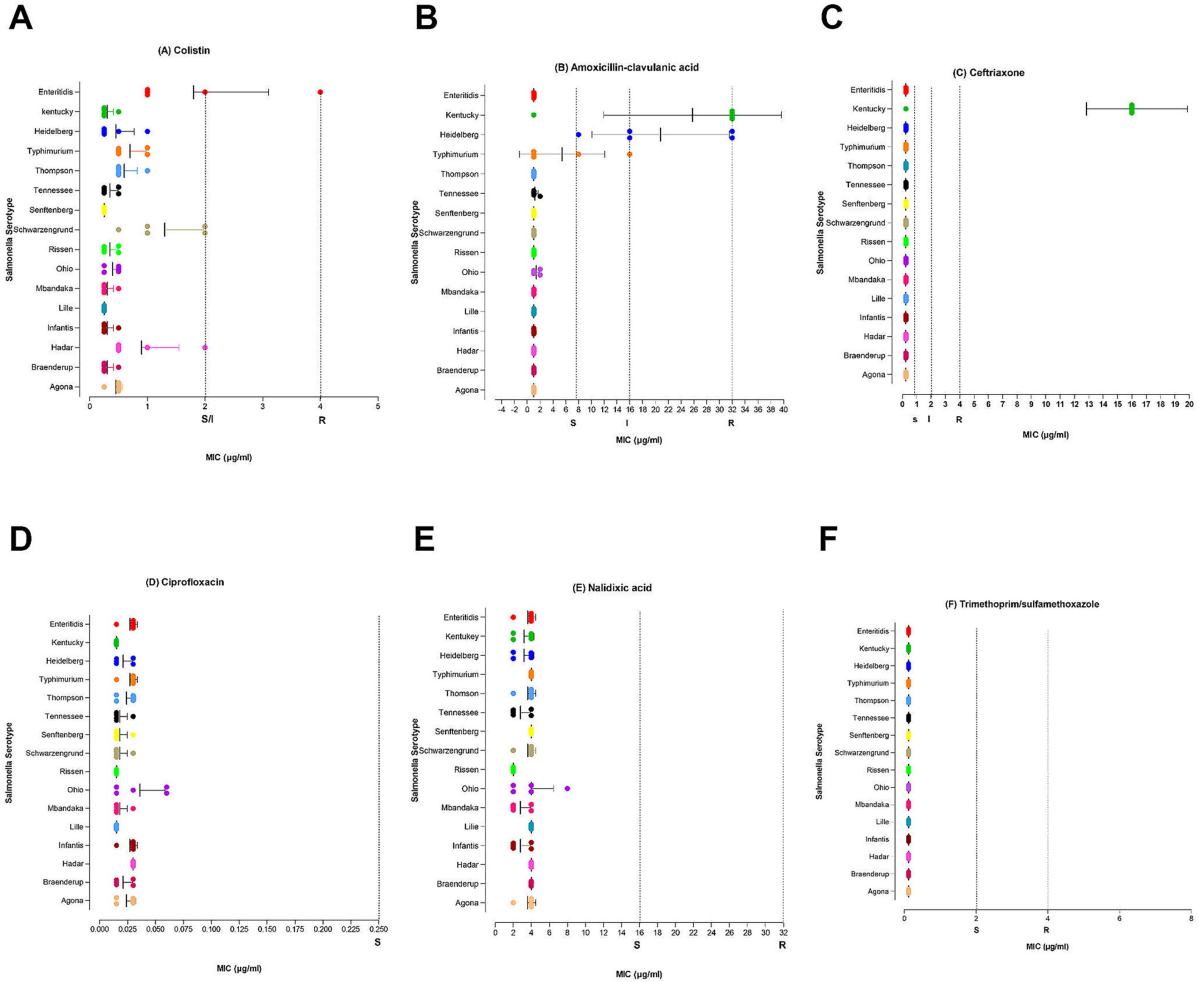
MIC distribution for category 1 antimicrobials at serovar level. Mean MIC values with bars representing standard deviation. **(A)** Colistin ≤ 2 μg/mL S and ≥ 2 μg/mL R. **(B)** Amoxicillin-clavulanic acid (≤ 8 / 4 μg/mL S, 16/8 μg/mL I, ≥ 32 / 16 μg/mL R). **(C)** Cefriaxone (≤1 μg/mL S, 2 μg/mL I, ≥ 4 μg/mL R). **(D)** ciprofloxacin (≤0.25 μg/mL S, 0.5 μg/mL I, ≥ 1 μg/mL R). **(E)** Nalidixic acid (≤16 μg/mL S, ≥ 32 μg/mL R). **(F)** Trimethoprim/sulfamethoxazole (≤2/38 μg/mL S, 4/76 ≥ 4 μg/mL R).

The minimum inhibitory concentration (MIC) distribution pattern for category 1 antimicrobials is shown in Figure 2. We observed only one *S. enteritidis* isolate that was phenotypically resistant to colistin (MIC = 4 μg/mL). Three *S. Kentucky* and 2 *S.* Heidelberg were resistant to amoxicillin-clavulanic acid (MIC ≥32 μg/mL). The same three *S. Kentucky* isolates were also resistant to ceftriaxone (MIC 16 μg/mL). All *Salmonella* isolates were susceptible to meropenem (MIC <=0.06 μg/mL), nalidixic acid (<= μg/ml), ciprofloxacin (<=0.015 μg/mL) and Trimethoprim/sulfamethoxazole (<=0.12 μg/mL).

**Figure 3:**
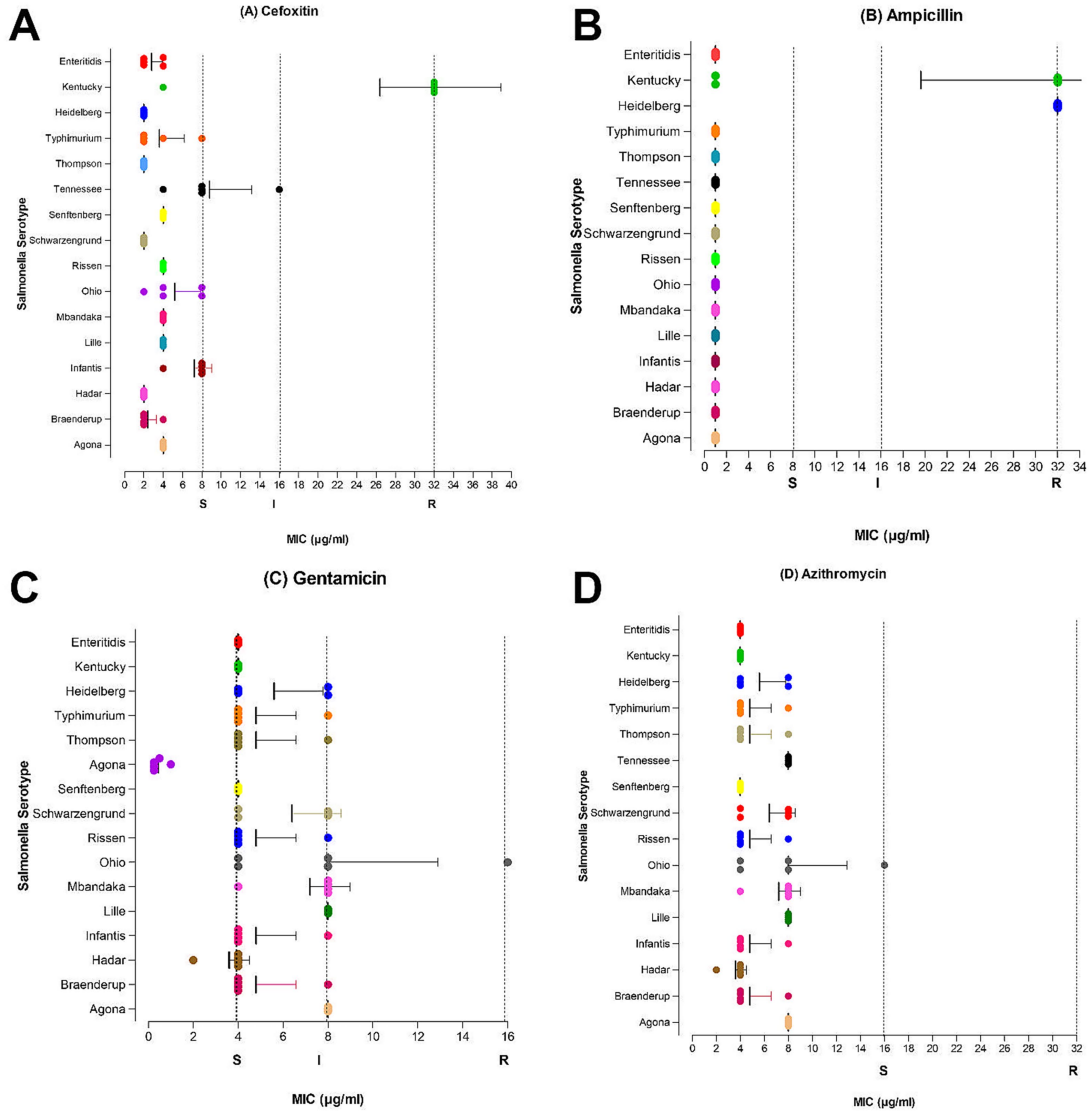
MIC distribution for category 2 antimicrobials at serovar level. Mean MIC values with bars representing standard deviation **(A)** Cefoxitin (≤8 μg/mL S, 16 μg/mL I, ≥32 μg/mL R). **(B)** Ampicillin (≤8 μg/mL S, 16 μg/mL I, ≥32 μg/mL R). **(C)** Gentamicin (≤4 μg/mL S, 8 μg/mL I, ≥16 μg/mL R). **(D)** Azithromycin (≤16 μg/mL S, ≥32 μg/mL R).

For category 2 antimicrobials (Figure 3) resistance was detected in two drugs: cefoxitin (>32 μg/mL) and ampicillin (>32 μg/mL). Cefoxitin and ampicillin resistance were observed in those three isolates of ser. Kentucky mentioned above. Moreover, all *S.* Heidelberg (>32 μg/mL) isolates and one *S. typhimurium* (>32 μg/mL) were phenotypically resistant to ampicillin, which warrants public health attention to prevent further dissemination. All isolates were found susceptible to azithromycin (<= 16 μg/mL) and gentamicin (<= 0.5 μg/mL). Resistance to category 3 antimicrobial drug tetracycline was observed in 21% of the *Salmonella* isolates (≥32 μg/mL). Only one *S. typhimurium* isolate was resistant to Sulfisoxazole (>256 μg/mL) and two were resistant to florfenicol (>32 μg/mL) (Category 3 antibiotics data is not shown).

**Figure 4:**
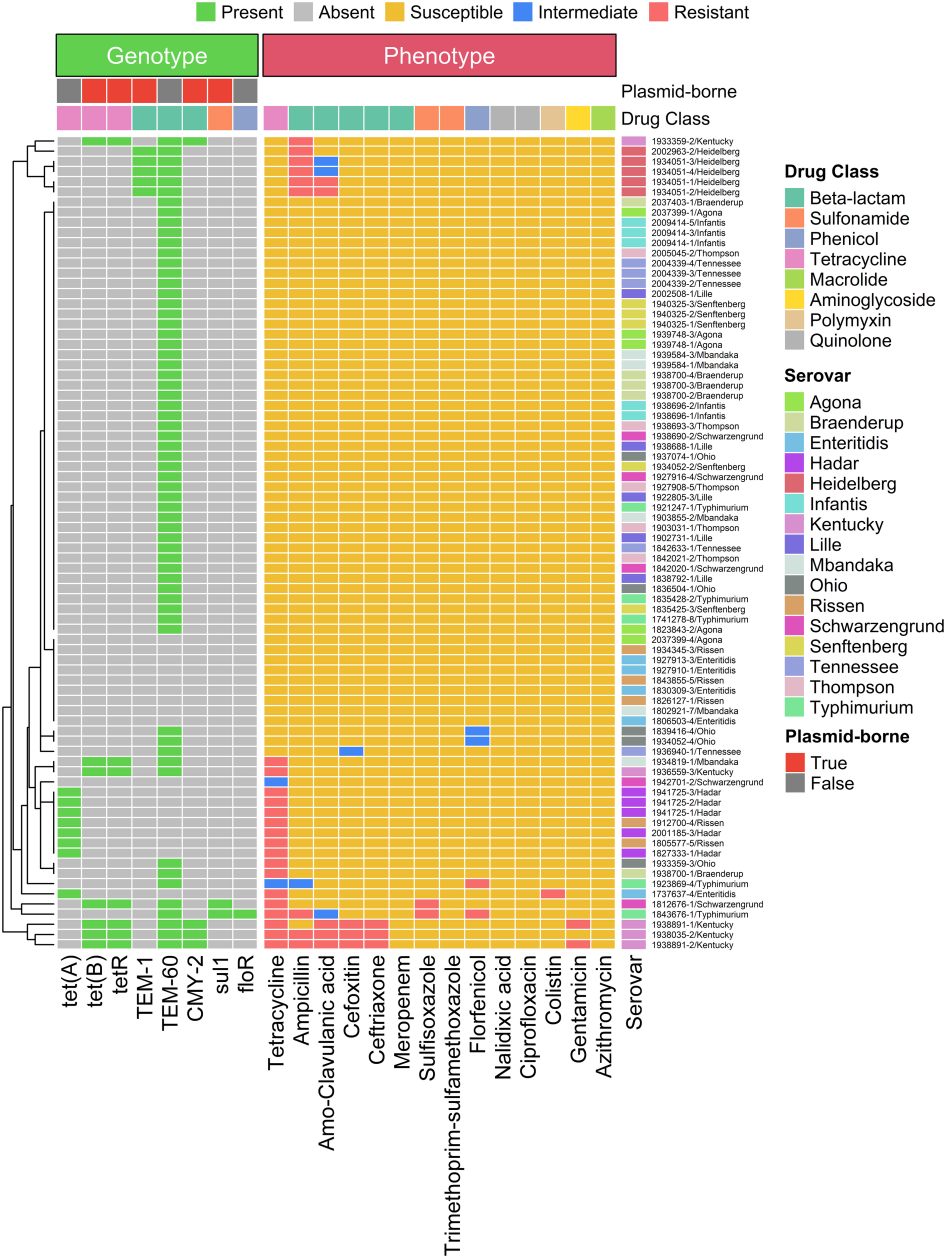
Comparison of antimicrobial susceptibility genotype and phenotype of the *Salmonella* isolates to the different antimicrobial tested. The AMR genes located in plasmids are indicated in a separate panel.

Figure 4 summarizes a side-by-side comparison between the AMR genotypes of each isolate and their antimicrobial susceptibility profiles. Since only a small subset of isolates exhibited AMR by phenotypic testing, the figure illustrates selected genes that may explain acquired resistance; numerous efflux pump genes conserved across all serovars are not shown. Colistin, a last-resort antimicrobial, showed resistance in one *S. enteritidis* isolate; however, no known resistance genes associated with colistin resistance were detected. Extended-spectrum beta-lactamase (ESBL) genes, TEM-1 and CMY-2, were detected in *S.* Heidelberg and *S.* Kentucky isolates. The presence of these genes correlated with resistance phenotypes to multiple beta-lactam antimicrobials, including amoxicillin–clavulanic acid, ceftriaxone, and cefoxitin. Notably, most serovars harbored TEM-60, an ESBL, irrespective of their resistance phenotype. Another positive phenotype–genotype correlation was also observed for sulfonamides: all sulfisoxazole-resistant isolates (*n* = 2) carried the sul1 gene. Similarly, one florfenicol-resistant *S. typhi*murium isolate carried the floR gene. More than half of the tetracycline-resistant isolates carried tetA, while the remainder carried tetB and/or tetR. Although not illustrated in the figure, the majority of isolates harbored multiple efflux pump genes regardless of serovar or resistance phenotype. We further observed that tet(B), tetR, CMY-2, TEM-1, and sul1 were plasmid-associated, distributed across *S.* Kentucky, *S. typhi*murium and *S.* Schwarzengrund.

### Detection of the *Salmonella* gene-encoding virulence factors

The major *Salmonella* virulence factors of interest were categorized into attachment factors, iron uptake systems, and effector proteins associated with Type 3 secretion system (T3SS) encoded in *Salmonella* pathogenicity island 1 (SPI-1) and 2 (SPI-2). Genes associated with bacterial attachment included type I fimbriae (*fimC, fimF, fimH, fimI*), Curli fimbriae (*csgA*, *csgB*, *csgC*, *csgE*, *csgF*, *csgG*), long polar fimbriae (*IpfA, IpfB, IpfC, IpfD, IpfE,*), plasmid encoded fimbriae (*pefA, pefB, pefC, pefD*), and F4 fimbriae (*faeC, faeD, faeE*). Genes encoding Type 1 fimbriae were detected in all isolates irrespective of serovar except for one isolate in *S.* Hadar. Curlie fimbriae were universally present in all isolates. Except for *S.* Ohio and Schwarzengrund, all isolates carried long polar fimbriae. Plasmid-encoded fimbriae were exclusively present in *S. enteritidis* isolates and two isolates of *S. typhi*murium, while F4 fimbriae were only detected in *S.* Thompson, Lille, Schwarzengrund, and Kentucky. It is well established that fimbriae diversity within the *Salmonella* genome determines host range, with host-adapted lineages (e.g., *S.* Dublin, *S. choleraesuis*, *S. typhi*) often exhibiting patterns of fimbriae degradation (Kisiela et al., 2012). Consistent with their broad host range, we found that *S. enteritidis* and *S. typhimurium* harbor the highest number of fimbriae.

Siderophores act as ferric ion scavenging molecules for bacteria. Our data indicated a diversity in the distribution of key siderophore biosynthesis gene clusters among poultry isolates. The catecholate type siderophore known as enterobactin (encoded by *entABCDE*) is generally found in all *Salmonella* serovars. Enterobactin possesses a very high affinity to Fe^3+^ in nature. Absence of *entE* in all serovars except in *Salmonella* Tennessee suggested that enterobactin synthesis does not play key a role in poultry isolates for the colonization in the bird (in the gastrointestinal tract). The enterobactin molecule can be glycosylated by an enzyme called glycosyl transferase encoded by *iroB* to create salmochelin which has the same affinity for Fe^3+^ as enterobactin. The reason behind glycosylation of enterobactin is to evade lipocalin mediated impedance to ferric-enterobactin complex. Lipocalins are secreted by phagocytic cells during inflammation to sequester iron bound siderophores. As a result, bacteria will not be able to take up this ferric-siderophore. This is part of the host defense mechanism called nutritional immunity. Lack of enterobactin will result in absence of salmochelin, hence serovars lacking *entE* did not encode gene related to biosynthesis and uptake of salmochelin (*iroBCDE, iroN*). The only exception for this was *Salmonella* Kentucky which had intact biosynthesis and uptake of salmochelin amid lack of enterobactin production. Interestingly *Salmonella* Tennessee can produce enterobactin but lack synthesis of salmochelin and uptake. So, our data here suggested that salmochelin mediated iron uptake does not seem to be essential for colonizing chicken.

Aerobactin is a citrate-hydroxymate type siderophore, which is not typically found in *Salmonella*. Its biosynthesis is encoded by *iucABCD* and uptake mediated by a dedicated receptor, *Iut*. Among all the poultry isolates tested in this study, *Salmonella* Kentucky is the only serovar found to carry aerobactin synthesis genes. While rare, the emergence of *Salmonella enterica* strains encoding aerobactin synthesis and uptake genes has been documented, often through horizontal acquisition of the plasmid pColV (colicin V virulence plasmid) (Wellawa et al., 2020). Moreover, previous studies have shown that *E. coli* pathotypes carrying aerobactin exhibit enhanced pathogenicity (Torres and Payne, 1997). Aerobactin binds to ferric iron with high affinity, albeit less strongly than enterobactin or salmochelin. We therefore hypothesize that, in *S*. Kentucky strains, aerobactin complements enterobactin-mediated ferric uptake, potentially providing a fitness advantage that supports clonal expansion in poultry production environments.

**Figure 5:**
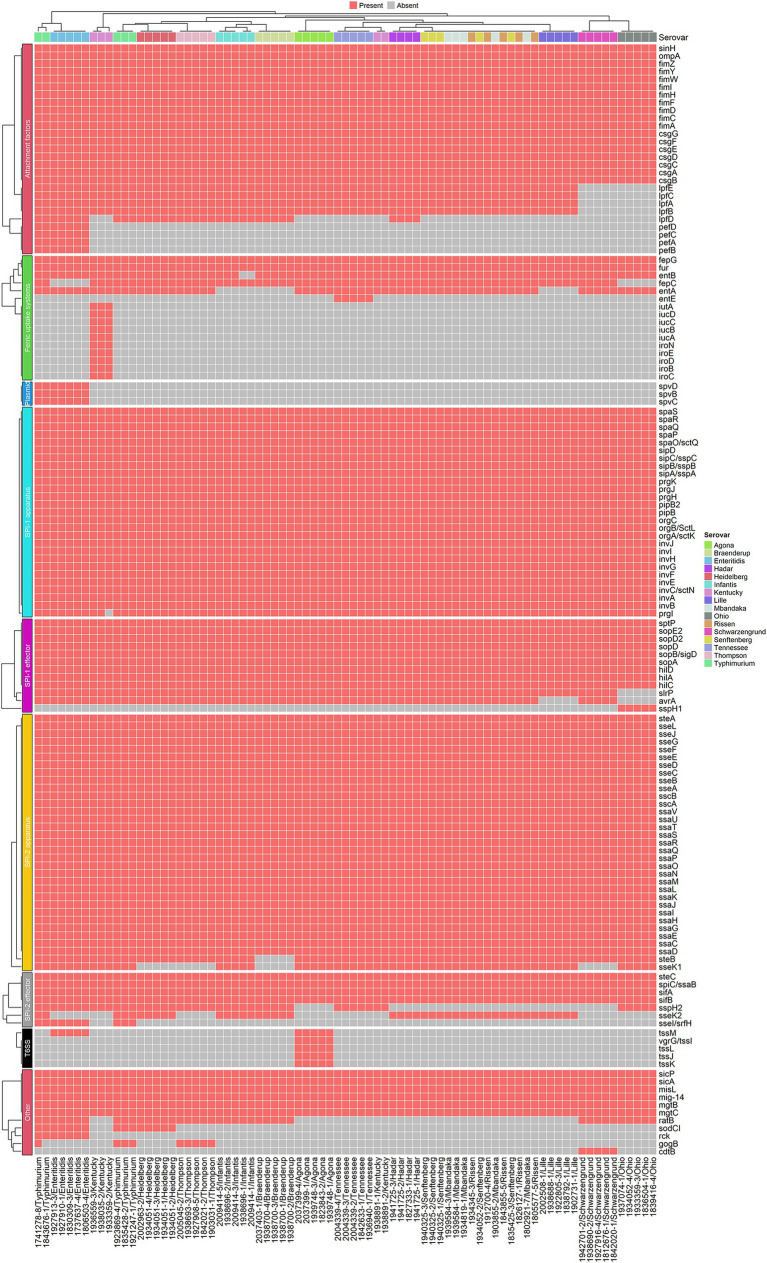
Distribution of gene-encoding virulence factors across the *Salmonella* isolates (*N* = 80) examined in the study. The isolates are clustered according to their virulence gene presence and absence profiles. A presence of a gene is determined when the sequence identity and alignment coverage between the reference and the tested isolate is >80%.

Genetic markers encoding T3SS effector proteins in SPI-1 were universally distributed among all isolates, as shown in Figure 5. The main components encoded on SPI1 include the subunits of a type III secretion apparatus [Cytoplasmic component: OrgA, SpaO, SpaR, SpaS, InvI; Export apparatus: SpaP, SpaQ, SpaR, SpaS, InvA; needle base: InvG, PrgI, PrgJ; needle filament: PrgI, Prg, effectors secreted by the apparatus, factors required for their efficient translocation (SipB, SipC, SipD), and transcriptional regulators (HilA, SprB, HilC (SprA/SirC), HilD and InvF)]. Moreover, they are involved in other activities including actin rearrangement for invasion (*sopE*/*sopE2*, *sopB*, *sopA*) (Lostroh and Lee, 2001; Cascales, 2017). Genes encoding effectors secreted through SPI-2 T3SS apparatus were present in all of the isolates tested (data not shown). These are involved in *Salmonella* containing vacuole maturation and positioning (*spvB*, *sseF*, *sseG*, *sseL*, *sspH2*), Sif extension (*pipB2*, *sifA*, *sopD2*), host cell signaling (*sseL*, *spvC*), induction of apoptosis (*spvB*, *sseL*). Two virulent genes, *spv*A, *spv*B are arranged as an operon in a plasmid and translocated to cell via SPI2- T3SS (Guiney and Fierer, 2011). These effectors directly involve in regulation of cell apoptosis and were mostly present in our *Salmonella Enteritidis* isolates.

### Phylogenetic analysis of Canadian *Salmonella* serovars from nanopore WGS data

**Figure 6:**
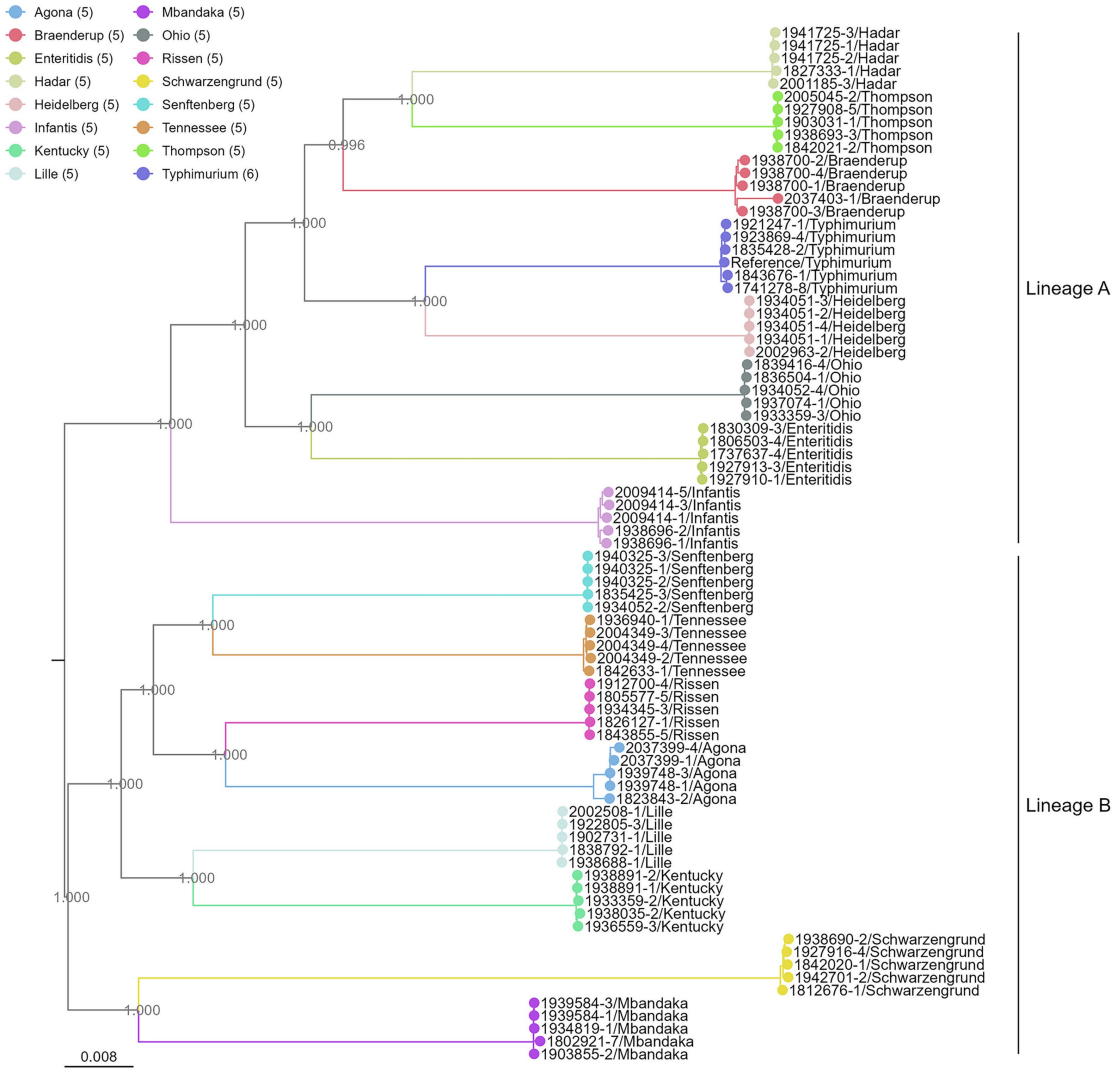
Phylogenetic analysis of the 80 *Salmonella* isolates tested. The maximum likelihood phylogeny of the isolates was constructed from concatenated core genome SNV alignment using RAxML and rooted by an *S. bongori* isolate (NCBI accession ID GCF_002952975.1). The branch length in the tree is defined as the number of single nucleotide substitutions per site. Sites correspond to positions along the variable segment (polymorphic regions) of the *Salmonella* core genome. Bootstrap supports are shown in the internal nodes of the tree.

Phylogenetic analysis of all Canadian isolates sequenced in this study revealed complete segregation by serovar identity. Isolates of the same serovar formed monophyletic clades with high clonality, indicating minimal intra-serovar variation within the province (Figure 6). Notably, several serovar pairs are depicted as sister clades, the relationships of which are consistent with previous reports (Timme et al., 2013; Worley et al., 2018). As expected, well-characterized orthologous serovars, such as Rissen, Agona, Kentucky, Senftenberg, and Tennessee formed a distinct clade within the same lineage—Lineage B. Additionally, the tree topology highlighted a skewed distribution of human pathogens. The leading causative agents of global foodborne illnesses, including Enteritidis, Typhimurium, Heidelberg, and Infantis (Ferrari et al., 2019) are collectively grouped within Lineage A, suggesting a shared evolutionary origin of enhanced pathogenicity.

## Materials and methods

### Bacterial isolates selection and serotyping

Our study examined the 15 most prevalent serovars identified in poultry environments in Saskatchewan, Canada between 2017 and 2022. Five isolates per serovar were randomly selected from a biobank of 1,895 isolates previously characterized by our research group. Additionally, *S. enteritidis* were included due to its public health significance and relevance to the poultry industry. This resulted in a total of 80 *Salmonella* isolates, evenly distributed across 16 serovars. The prevalence data of the selected serovars in our region between 2017 and 2022 was as follows: *S*. Kentucky (*n* = 489, 25.80%), Mbandaka (*n* = 233, 11.77%), Infantis (*n* = 197, 10.40%), Tennessee (*n* = 108, 5.70%), Typhimurium (*n* = 101, 5.33%), Schwarzengrund (*n* = 98, 5.17%), Thompson (*n* = 82, 4.33%) Lille (*n* = 78,4.12%), Hadar (*n* = 72, 3.8%) Senftenberg (*n* = 70, 3.69%), Agona (*n* = 53, 2.80%), Braenderup (*n* = 53, 2.80%), Rissen (*n* = 45, 2.37%), Ohio (*n* = 31, 1.64%), Heidelberg (*n* = 25, 1.32%) and Entertidis (*n* = 14, 0.74%).

To establish the ground truth serovar assignments, all isolates were sent to the OIE Reference Laboratory for Salmonellosis in Guelph, Ontario for serotyping by sequencing, using the Illumina platform. Prior to serotyping, all suspected colonies were confirmed to be *Salmonella* by sub-culturing on Colombia sheep blood agar plates, followed by slide agglutination test with Salmonella specific Poly-O antiserum and the Matrix-Assisted Laser Desorption Ionization Time-of-Flight Mass Spectrometry (MALDI-TOF MS, Bruker Daltonics, Billerica, MA).

### Antimicrobial susceptibility testing of *Salmonella* isolates

Antimicrobial susceptibilities of the 80 isolates were determined using the microbroth dilution method as per Clinical Laboratory Standard Institute (CLSI) guidelines. The commercially available Sensititre™ NARMS Gram Negative CMV4AGNF AST Plates (SensititreTM Trek Diagnostic Systems Ltd., West Sussex, UK) were used. The plates consisted of critically important category I antimicrobial drugs (amoxiciliin clavulanic acid, ceftriaxone, ciprofloxacin, colistin, meropenem, nalidixic acid), category II (Ampicillin, azithromycin, cefoxitin, gentamicin, trimethroprim-sulfamethoxazole); and category III (tetracycline, sulfisoxazole and florfenicol) antimicrobial drugs. The minimum inhibitory concentrations (MIC) values were read using Sensititre Auto Reader. In the case of an antimicrobial combination such as trimethroprim-sulfa, the MIC of the first agent (trimethoprim) was reported as the MIC for the combination. Thereafter, the isolates were categorized as susceptible, intermediate, or resistant based on Clinical and Laboratory Standards Institute (CLSI)- MIC breakpoints (CLSI VET01S ED6:2023).

### Whole genome sequencing of *Salmonella* isolates using Oxford Nanopore Technology

DNA extraction from the bacterial isolate was performed using the Qiagen MagAttract HMW DNA Kit (48) (Qiagen, Cat. No. 67563) with a modified version of the manufacturer’s protocol (*Document version: Manual Purification of High-Molecular-Weight Genomic DNA from Gram-Negative Bacteria—Version 03/2020*). A third of a 10 μL loop of the bacterial isolate from the blood agar plate was suspended in 200 μ1 of lysis mix (180 μL PBS and 20 μL Proteinase K) and incubated at 56 °C for 30 min at 900 rpm. Following lysis, DNA extraction was carried out according to the manufacturer’s protocol from Step 3 onward. For elution 200 μL of nuclease-free water was added to the beads, followed by incubation at 37 °C with 900 rpm for 15 min. The extracted DNA was quantified using the Qubit 1X dsDNA High Sensitivity Assay Kit (Thermo Fisher Scientific, Cat. No. Q33231).

**Figure 7:**
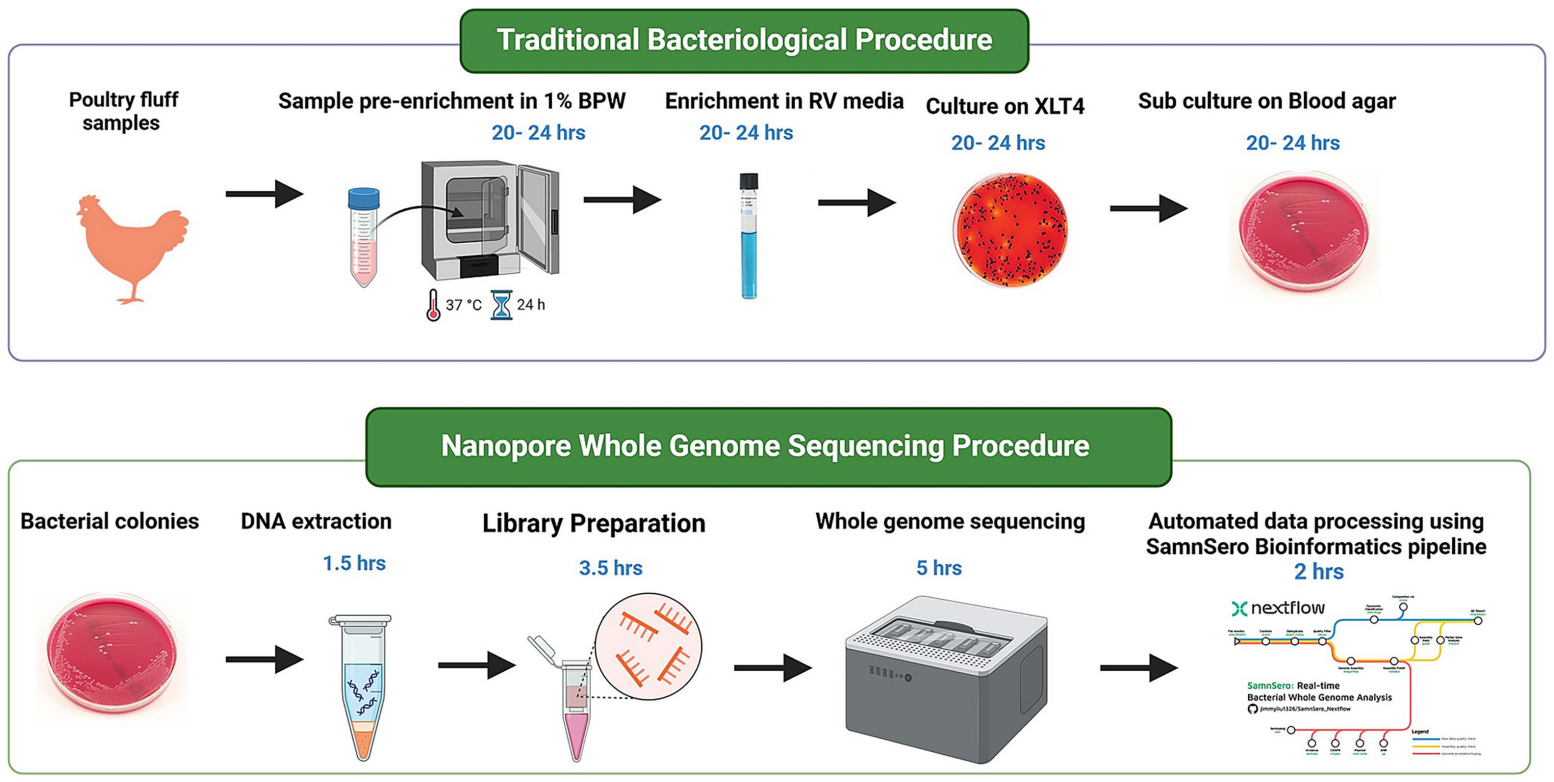
Flowchart illustrating *Salmonella* isolation from poultry fluff samples, followed by whole genome sequencing procedure from the bacterial pellet obtained from enrichment broth. Time estimates for each step are calculated based on batch processing of 12 samples.

The sequencing library was prepared using the Ligation sequencing gDNA - native barcoding (SQK-LSK109 with EXP-NBD104 and EXP-NBD114) protocol (*Document version: NBE_9065_v109_revAP_14Aug2019*). The extracted DNA was normalised to 1,000 ng and taken to end-preparation. The NEBNext Ultra II End repair/dA-tailing Module (NEB, Cat no. E7546) and NEBNext® FFPE DNA Repair Mix (NEB, Cat no. M6630L) were used to add dA-tails to the ends of the amplicons, followed by magnetic bead clean up and elution with 25 μL of nuclease free water. Subsequently, unique barcodes from EXP-NBD104 and EXP-NBD114 were ligated to the end-prepped amplicons using NEB Blunt/TA Ligase Master Mix (NEB, Cat no. M0367). After barcoding, samples with unique barcodes were cleaned up using magnetic beads and equi-molar amount of each barcode were pooled together. Sequencing adapters were then ligated to the pooled samples using the NEBNext Quick Ligation Module (NEB, Cat no. E6056), followed by magnetic bead clean up. Finally, the resulting library was quantified using Invitrogen Qubit 4 Fluorometer with the [Qubit 1x dsDNA HS Assay Kit (Thermofisher, Cat no. Q33231)]. The prepared library was loaded onto an R9.4 flow cell (FLO-MIN106 SpotON) and sequenced on a GridION sequencer until 150 Mb (30 × coverage) of data was generated for each sample. Basecalling was performed in real time using the high accuracy basecalling option (Guppy version 4.2.3) within MinKNOW (Version 20.10.6). The sequence data was then used for bioinformatic analysis (Figure 7). The cost estimates to complete a batch of 12 samples are indicated in Table 1.

**Table 1:** Cost estimate for consumables used for *Salmonella* isolation, DNA extraction and whole genome sequencing.

Bacteriological and WGS procedure steps involved	Cost estimate per 12 samples (CAD)
Sample pre-enrichment in 1% Buffered Peptone Water	2.4
Enrichment in Rappaport Vassiliadis broth	3.24
Culture on Xylose Lysine Tergitol 4 (XLT4) agar	14.16
Culture on Columbia Agar with 5% sheep blood	5.4
DNA extraction	574.93
DNA repair and End preparation	430.14
Native barcode ligation	469.06
Adaptor ligation and clean up	118.07
Loading to flow cells and sequencing	705.61
Total cost per 12 samples	2323.01

### *SamnSero*: an open-source bioinformatics pipeline to streamline the analysis of nanopore whole genome sequencing data

All WGS data analysis conducted in this study, excluding phylogenetic reconstruction, is packaged as an open-source software called “SamnSero” (accessible from https://jimmyliu1326/SamnSero_Nextflow). The software is implemented in Nextflow (Langer et al., 2025), a data analysis workflow engine widely adopted by the scientific community for orchestrating data processing in an efficient, scalable and reproducible manner. The SamnSero pipeline can be divided into three phases: pre-assembly processing, *de novo* genome assembly and post-assembly processing. Prior to genome assembly, sequences are first quality filtered and trimmed for quality control purposes. The process includes: barcode and adaptor trimming using Porechop (Bonenfant et al., 2023), read length (>1,000 bps) and read quality (>Q10) filtering using Nanoq (Steinig and Coin, 2022), and taxonomic composition estimation using Centrifuge (Kim et al., 2016) to detect cross-species contamination and identify the proportion of on-target (*Salmonella*) reads. Following the pre-processing steps, the quality filtered reads are assembled to generate an overlap layout consensus graph using a long-read assembler, Flye (Kolmogorov et al., 2020). Medaka,^1^ a genome polishing tool specifically optimized for correcting Flye assemblies is subsequently used to correct errors in draft genome assemblies. In the final phase of the pipeline, the polished assemblies are annotated to identify an array of genetic markers indicative of serovar identity, virulence and antimicrobial resistance. Specifically, *in silico* serotyping is conducted using Salmonella *In Silico* Typing Resource (SISTR) (Yoshida et al., 2016), the same software used by the local *Salmonella* OIE reference lab. Virulence factors are identified using Abricate^2^ based on 80% identity and 80% coverage of reference sequences of known virulence genes from VFDB (Liu et al., 2022). Antimicrobial resistance markers are identified using the Resistance Gene Identifier (RGI) that searches against the Comprehensive Antibiotic Research Database (CARD) (McArthur et al., 2013). To evaluate the mobility potential of the genetic risk factors, plasmids are identified using MOB-Suite (Robertson and Nash, 2018), which also categorizes identified plasmids by their mobility potential (non-mobilizable, mobilizable, conjugative) based on mobility markers, such as oriT, relaxases and MPF genes. Finally, to establish our confidence in the genomic results, the quality of the polished assemblies is assessed using QUAST (Gurevich et al., 2013) and CheckM (Parks et al., 2015) to report metrics such as contiguity (i.e., N50), completeness and contamination. To streamline the reporting of the genomic results to laboratory technologists, veterinary microbiologists and stakeholders, all of the results from the final phase of the pipeline are summarized and displayed in interactive HTML reports generated using RMarkdown (Holmes et al., 2021) (An example report can be viewed at https://jimmyliu1326.github.io/SamnSero_Nextflow/assets/qc_report.html). Furthermore, we have taken steps to make the software more accessible to non-technical users by supporting compatibility of SamnSero with EPI2ME—a bioinformatics desktop application developed by ONT. This added compatibility enables non-technical users to operate complex bioinformatics tools through a more user-friendly, intuitive graphical interface.

### Phylogenetic analysis of *Salmonella* serovars

*Salmonella* genome assemblies were aligned to the *S. typhimurium* LT2 strain reference genome (NCBI accession ID GCF_002952975.1) using nucmer provided by the MUMmer3 software suite (Kurtz et al., 2004) to identify single nucleotide polymorphisms (SNPs). The SNP positions were concatenated into a multiple sequence alignment representing all polymorphic sites (excluding gaps) in the *Salmonella* genome. The concatenated alignment was supplied as input to RAxML (Stamatakis, 2014) to construct a maximum likelihood tree with 1,000 bootstrap replicates using the GTR model and gamma distribution for rate heterogeneity. The phylogeny was rooted using a *Salmonella bongori* genome (NCBI accession ID NCTC 12419) and visualized in RStudio using the ggtree R package (Yu et al., 2017).

## Discussion

Despite the common perception of NS yielding higher sequencing error rates compared to established incumbents in the sequencing space, our data demonstrated that the long-read platform can produce identical *Salmonella* serovar predictions to those generated by short-read sequencing. This successful replication underscores the growing maturity of NS in bacterial genome reconstruction, which can be attributed to recent advancements in nanopore sequencing accuracy. Previous studies have shown that NS at 30x coverage is sufficient to achieve high-quality (>Q30; 99.9% accuracy) bacterial genome and plasmid assemblies (Kovaka et al., 2023). Recent developments, such as the release of the R10.4.1 flowcell and the ligation kit V14, have further reduced ONT error rates by addressing indel errors that were notoriously problematic in homopolymeric regions. Sanderson et al. (2024) recently reported the construction of near-finished *E. coli* assemblies using the latest nanopore chemistry and the most up-to-date Dorado basecalling model, the combination of which in fact outperformed the accuracy of hybrid genome assemblies. The increasing accuracy of single pass nanopore reads have also led to increasing approvals of NS for tracing bacterial outbreaks, where related cases can differ by as little as one nucleotide (Lohde et al., 2024). This newfound capacity to accurately subtype clonal bacterial isolates of the same outbreak has been attributed to ONT’s updated DNA basecalling model which corrected base substitution errors frequently induced by native base modifications, i.e., methylation (Sanderson et al., 2024). As sequencing accuracy continues to improve, the required sequencing depth for accurate bacterial genome assemblies is expected to decrease, leading to further reductions in sequencing costs—an important factor in resource-limited settings (Mostafa, 2024).

Using our protocol, we successfully multiplexed up to 12 *Salmonella* isolates on a single MinION flowcell producing sufficient sequencing reads to conduct comprehensive Whole Genome Sequencing (WGS) analyses. Subsequent quality assessment of the resulting assemblies highlighted the strengths of NS, yielding highly contiguous and complete bacterial assemblies with minimal evidence of contamination. It is worth noting that we only evaluated genome assembly quality strictly based on raw assembly statistics and species marker genes. Evaluating the accuracy of bacterial assemblies at the nucleotide level is beyond the scope of this study. For a more comprehensive benchmark between NS and Illumina sequencing in bacterial assembly reconstruction, we refer the readers to the following studies (Sanderson et al., 2024; Lerminiaux et al., 2024). Building on the high-quality bacterial genome reconstructions, we streamlined the generation of a *Salmonella* risk matrix composed of serotyping, phylogenetic inference, and genetic determinants of AMR and virulence. The successful replication of gene annotation and phylogenetic outputs in accordance with previous reports and phenotypic testing, further highlighted the reliability of our NS-based *Salmonella* WGS protocol. Our protocol can be leveraged by other diagnostic laboratories to build up local sequencing capacity, enabling comprehensive diagnostic information to be delivered to stakeholders and veterinary microbiologists within less than 48 h after culturing. This approach offers the potential of achieving a significantly faster turnaround time than what is publicly and commercially available in Canada. From a financial perspective, the lower cost of entry (equipment cost) and per sample sequencing cost (approximately $200 CAD per isolate using MinION) empower other laboratories to readily implement and reproduce our end-to-end workflow, paving the path for efficient and comprehensive *Salmonella* risk assessments on a broader scale. By broadening access to sequencing-based solutions, we aim to advocate for the democratization of sequencing, helping to establish a much-needed decentralized model for *Salmonella* diagnostics and surveillance.

Accompanying our NS protocol is a comprehensive *Salmonella* genomic analysis toolkit. We have bundled all the necessary data analysis tools for processing nanopore sequencing data into a single open-source software package, implemented in Nextflow to ensure scalability, portability, and reproducibility (see Materials and Methods). Users can run the entire data analysis workflow—from data quality assessment and filtering to *de novo* genome assembly, genome annotation, and *in silico* serotyping—directly from a unified graphical interface provided by EPI2ME. Through this tool, non-technical users can interact with the analysis workflow in a simple point-and-click manner, enabling complex bioinformatics tasks to be performed with minimal training. We have also streamlined the reporting of genomic results, including sequence data quality, serotyping, and gene predictions, which are integrated into shareable HTML reports that can be saved locally and distributed to stakeholders over the web.

Multidrug resistant *S*. Kentucky ST198 lineage, originating from poultry products, has been implicated to be a key source of many enteric outbreaks globally. Indeed, our results demonstrate that these MDR strains can be detected in the Canadian poultry environment (Card et al., 2023). Additionally, the detection of colistin resistance in poultry raises concern, given colistin’s use as a last-resort antibiotic for gram-negative infections. Colistin resistance has been reported to be primarily driven by the presence of plasmid-associated genes, such as mcr-1,2,3,4,5,9 (Gogry et al., 2021) or the acquisition of point mutations in *pmrB* (Haeili et al., 2018), all of which were not detected in our resistant isolate. Further investigation is needed to determine whether this insensitivity is due to resistance mediated by an undocumented mechanism or the presence of a more distant homolog that requires adjustments to our search algorithm. This finding also underscores the need for continued surveillance of colistin resistance, particularly in the poultry production environment, to identify risk factors and mitigate its potential impact on public health and food safety.

On the other hand, we have identified scenarios where the isolates were genotypically resistant, but phenotypically susceptible. For example, numerous isolates harboring aminoglycoside-modifying genes presented a susceptible phenotype to aminoglycosides. It is possible that while resistant genes may be present, they may not be actively expressed within the bacteria and therefore no resistance is conferred. Similarly, we identified many isolates harboring a large repertoire of efflux pumps targeting beta-lactams, aminoglycosides, fluoroquinolones and macrolides, yet exhibiting susceptibility to these antibiotics. Hence, we reasoned that AMR genotype–phenotype discrepancies could also arise from basal expression of AMR genes being insufficient to manifest resistance. Overexpression of efflux pumps, potentially regulated by environmental cues, or the acquisition of additional gene copies may be necessary to meet resistance breakpoints. As such, accurately predicting antibiotic susceptibility from genotypic data remains a significant challenge. However, our NS protocol provides a robust foundation for obtaining high quality genotypic data, which can support ongoing community efforts to improve the curation of AMR knowledge bases. Progressing our understanding of the molecular mechanisms of AMR will help us move closer to establishing data-driven expert rules that standardize the interpretation of antibiotic susceptibility from genotypic data.

Bacterial pathogens continuously modify their genomic information via gene exchange or random mutations. These genomic changes can alter bacterial pathogenicity and transmissibility, leading to the emergence of novel variants with epidemic potential. Among the key determinants of *Salmonella* pathogenicity, we detected remarkable diversity in fimbrial adhesins, which are critical for bacterial attachment to surfaces, such as the intestinal epithelium (Cheng and Wiedmann, 2021). Given its important role in mediating host-pathogen interactions, diverse fimbrial structures expressed by *Salmonella* have been extensively studied to elucidate their roles in driving host and tissue tropism (van Asten and van Dijk, 2005). Through NS, we revealed diversity among plasmid-encoded fimbriae and F4 fimbriae, with their distributions correlated with serovar classifications. For example, plasmid-encoded fimbriae were exclusively present in *S. entertidis* and *S. typhi*murium, potentially augmenting *Salmonella* pathogenicity. Both *S. enteritidis* and *S. tyhimurium* are common causative agents of invasive NTS infections, previously attributed to their acquisition and maintenance of an expanded repertoire of fimbrial adhesins (Marchello et al., 2021). As distinct fimbrial adhesins facilitate attachment to specific host tissues and cell types, they play a pivotal role in driving systemic infections and more severe clinical outcomes, such as septicemia. Hence, monitoring the molecular evolution of bacterial adhesins could provide valuable insights on the health risks of NTS and enable early detection of hypervirulent strains.

Once *Salmonella* attaches itself to the gut epithelium, T3SS genes encoded on SPI-1 are expressed leading to the formation of a molecular syringe that transports effector proteins into the host cells. The effector proteins then act to trigger conformational changes that alter the host membrane, thereby facilitating endothelial uptake and invasion (Wellawa et al., 2020). Consistent with the vital nature of the T3SS in *Salmonella* pathogenesis, our data demonstrated that genes encoding the structural apparatus of SPI-1 T3SS were conserved across all serovars. Once internalized into host cells, the *Salmonella* containing vacuole is formed leading to the expression of a second T3SS encoded in SPI-2 (SPI-2 T3SS), which plays a critical role in causing systemic infections and intracellular pathogenesis. Similar to SPI-1 genes, the effector and structural proteins encoded on SPI-2 were also highly conserved across all isolates irrespective of serovars. With the exception of *SspH1*, *SspH2* and *Ssel*, these virulence genes were exclusively found in the genomes of *S. enteritidis*, *S. typhi*murium and *S.* Kentucky, suggesting their potential role in enhancing virulence. Past experiments demonstrated that *Salmonella sseL* mutant strains did not show a replication defect or induce altered levels of cytokine production upon infection of macrophages but caused a delayed cytotoxic effect, resulting in attenuated virulence in mice models. Another key mechanism of bacterial pathogenesis involves the dysregulation of host signaling pathways. SspH effectors are known to compete with host proteins for ubquitin conjugates, allowing Salmonella effector proteins to target host proteins involved in inflammatory signaling pathways that ultimately alter host immune response to favour its intracellular survival (Cook et al., 2019). These findings strengthen our confidence in the ability of NS to detect both variable and conserved virulence mechanisms that underline the broad spectrum of pathogenic potential across *Salmonella* serovars.

*Salmonella* utilizes iron sequestering siderophores for intracellular survival to rapidly colonize the gut and cause systemic dissemination. Enterobactin, in particular, is a widespread iron-chelating agent that is produced by gram-negative bacteria. Enterobactin biosynthesis genes were present in all serovars. However, genes involved in the biosynthesis of two other classes of siderophores, salmochelin and aerobactin, were only found in *S.* Kentucky, a major colonizer of the poultry gut. This may be one reason why *S*. Kentucky is dominated than *S. enteritidis* in a poultry environment as we have observed in this study. It is also hypothesized that if immunity directed against surface antigens of *S*. Entertidis due to continuous vaccination or exposure, the possibility of other serovar get upper hand (Foley et al., 2011). Moreover, acquisition of virulent plasmic from Avian pathogenic *E. coli* also lead *S*. Kentucky to increase the survival fitness in the microbial communities (Johnson et al., 2010). Our observation is consistent with previous reports that aerobactin production is primarily present among human salmonellosis cases linked to contaminated poultry products. This is likely due to ColV plasmids carried by *Salmonella* strains isolated from poultry, which encode Fe^3+^ uptake systems via synthesis, secretion and translocation of aerobactin (Wellawa et al., 2020). Enterobactin can be glycosylated by a glycosyltransferase enzyme, IroB, to form salmochelin which acts as a better iron scavenger than enterobactin (Wellawa et al., 2020). Through specialized modifications, salmochelin more effectively evades Lcn-2-mediated chelation, leading to enhanced bacterial survival and proliferation (Wellawa et al., 2020). Similar to aerobactin, salmochelin biosynthesis genes were present in select serovars, namely in *S.* Kentucky and *S.* Mbandaka. Salmochelin synthesis and transport require *iro* genes, *iroBCDE* and *iroN* in addition to enterobactin biosynthesis genes (Mey et al., 2021). Despite the key role of salmochelin in facilitating bacterial survival in iron-deficient environments, it remains unclear why majority of the serovars examined herein lack salmochelin biosynthesis genes. Overall, *S.* Kentucky isolates carried the highest number of siderophore biosynthesis genes which may explain its higher fitness in poultry. Our NS workflow thus lays the foundation for expanding our comparisons of siderophore-associated genes across a larger collection of strains and serovars, which could prove valuable in progressing our understanding of how host-pathogen interactions drive the selection of siderophores and ultimately contribute to the ecological success of select serovars.

By searching for known conserved elements critical to *Salmonella* pathogenesis, antigen biosynthesis and AMR, we demonstrate that nanopore genome assemblies at 30X coverage can provide valuable insights to genetic differences that underline microbial characteristics, behaviors and ancestry. From successfully reproducing OIE Reference Laboratory serotyping results to identifying the diverse spectrum of fimbrial adhesions associated with host and tissue tropism, our results collectively suggest that NS is a rapid, reliable and cost-effective tool for *Salmonella* molecular diagnostics and surveillance. As a natural extension, we are currently exploring the use of this technology to develop a metagenomic sequencing workflow to identify *Salmonella* serovars and related AMR and virulence markers directly from poultry samples. This approach will bypass the time-consuming process of bacterial culturing, offering a rapid alternative for addressing highly urgent diagnostic needs.

## Data Availability

The original contributions presented in the study are publicly available. The sequencing data generated from this study has been deposited in BioProject PRJNA1337272.

## Footnotes

^1^ https://github.com/nanoporetech/medaka

^2^ https://github.com/tseemann/abricate
