# Supplementary material for: Towards decentralization of Salmonella serotyping and risk assessment in poultry production environments with nanopore sequencing
Source: Front Microbiol. 2025 Oct 15;16:1669089. doi: 10.3389/fmicb.2025.1669089 (PMC12568515; doi:10.3389/fmicb.2025.1669089)
Supplement: Supplementary file 1 [file Table_1.docx]

| **Isolate ID** | **Serovar ID**  **(this study)** | **Serovar ID**  **(PHAC)** | **Genome Size (Mbps)** | **Completeness (%)** | **Contamination**  **(%)** | **No. of contigs** | **N50**  **(Mbps)** | **BioSample Accession** |
| --- | --- | --- | --- | --- | --- | --- | --- | --- |
| 1933359-2 | Kentucky | Kentucky | 5.074183 | 99.64 | 1.76 | 4 | 4.779535 | SAMN52211692 |
| 1936559-3 | Kentucky | Kentucky | 4.918231 | 99.26 | 1.76 | 2 | 4.771405 | SAMN52211702 |
| 1938035-2 | Kentucky | Kentucky | 5.058858 | 99.59 | 1.77 | 4 | 4.767016 | SAMN52211705 |
| 1938891-1 | Kentucky | Kentucky | 5.207013 | 99.48 | 1.29 | 5 | 4.799382 | SAMN52211715 |
| 1938891-2 | Kentucky | Kentucky | 5.244717 | 99.41 | 1.41 | 6 | 4.806727 | SAMN52211716 |
| 1802921-7 | Mbandaka | Mbandaka | 4.815617 | 98.47 | 0.82 | 5 | 3.158803 | SAMN52211662 |
| 1903855-2 | Mbandaka | Mbandaka | 4.719487 | 99.57 | 0.47 | 1 | 4.719487 | SAMN52211683 |
| 1934819-1 | Mbandaka | Mbandaka | 5.008697 | 99.24 | 0.66 | 2 | 4.68995 | SAMN52211701 |
| 1939584-1 | Mbandaka | Mbandaka | 4.917 | 99.27 | 0.5 | 1 | 4.917 | SAMN52211717 |
| 1939584-3 | Mbandaka | Mbandaka | 4.928232 | 99.27 | 0.5 | 3 | 4.916951 | SAMN52211718 |
| 1938696-1 | Infantis | Infantis | 4.728129 | 98.64 | 0.72 | 1 | 4.728129 | SAMN52211709 |
| 1938696-2 | Infantis | Infantis | 4.728022 | 98.91 | 0.46 | 1 | 4.728022 | SAMN52211710 |
| 2009414-1 | Infantis | Infantis | 4.727874 | 98.71 | 0.32 | 1 | 4.727874 | SAMN52211734 |
| 2009414-3 | Infantis | Infantis | 4.728205 | 98.45 | 0.58 | 1 | 4.728205 | SAMN52211735 |
| 2009414-5 | Infantis | Infantis | 4.697885 | 98.73 | 0.58 | 1 | 4.697885 | SAMN52211736 |
| 1842633-1 | Tennessee | Tennessee | 4.832605 | 98.53 | 0.99 | 1 | 4.832605 | SAMN52211678 |
| 1936940-1 | Tennessee | Tennessee | 4.863412 | 99.47 | 0.84 | 1 | 4.863412 | SAMN52211703 |
| 2004349-2 | Tennessee | Tennessee | 4.862824 | 98.81 | 0.78 | 1 | 4.862824 | SAMN52211730 |
| 2004349-3 | Tennessee | Tennessee | 4.871503 | 98.84 | 1.05 | 2 | 4.819306 | SAMN52211731 |
| 2004349-4 | Tennessee | Tennessee | 4.862854 | 98.77 | 0.81 | 1 | 4.862854 | SAMN52211732 |
| 1741278-8 | Typhimurium | Typhimurium | 4.985264 | 99.41 | 0.78 | 2 | 4.891258 | SAMN52211661 |
| 1835428-2 | Typhimurium | Typhimurium | 4.908866 | 99.49 | 0.41 | 3 | 4.88956 | SAMN52211672 |
| 1843676-1 | Typhimurium | Typhimurium | 5.031367 | 99.44 | 0.78 | 5 | 4.925185 | SAMN52211679 |
| 1921247-1 | Typhimurium | Typhimurium | 4.933667 | 99.00 | 0.41 | 7 | 4.889186 | SAMN52211684 |
| 1923869-4 | Typhimurium | Typhimurium | 4.925332 | 99.19 | 0.52 | 17 | 1.198973 | SAMN52211687 |
| 1812676-1 | Schwarzengrund | Schwarzengrund | 4.911266 | 99.43 | 0.64 | 2 | 4.640972 | SAMN52211665 |
| 1842020-1 | Schwarzengrund | Schwarzengrund | 4.696236 | 98.08 | 0.48 | 7 | 0.98762 | SAMN52211676 |
| 1927916-4 | Schwarzengrund | Schwarzengrund | 4.686011 | 98.82 | 0.8 | 1 | 4.686011 | SAMN52211691 |
| 1938690-2 | Schwarzengrund | Schwarzengrund | 4.700448 | 98.70 | 0.8 | 2 | 4.687936 | SAMN52211707 |
| 1942701-2 | Schwarzengrund | Schwarzengrund | 4.793936 | 95.76 | 0.53 | 17 | 0.779861 | SAMN52211727 |
| 1842021-2 | Thompson | Thompson | 4.710493 | 99.20 | 0.39 | 1 | 4.710493 | SAMN52211677 |
| 1903031-1 | Thompson | Thompson | 4.812165 | 99.58 | 0.41 | 2 | 4.764797 | SAMN52211682 |
| 1927908-5 | Thompson | Thompson | 4.764885 | 99.48 | 0.39 | 1 | 4.764885 | SAMN52211688 |
| 1938693-3 | Thompson | Thompson | 4.710685 | 99.17 | 0.39 | 1 | 4.710685 | SAMN52211708 |
| 2005045-2 | Thompson | Thompson | 4.76482 | 99.51 | 0.39 | 1 | 4.76482 | SAMN52211733 |
| 1838792-1 | Lille | Lille | 4.994535 | 99.37 | 1.1 | 2 | 4.985938 | SAMN52211674 |
| 1902731-1 | Lille | Lille | 4.994381 | 99.29 | 1.09 | 2 | 4.985746 | SAMN52211681 |
| 1922805-3 | Lille | Lille | 4.984045 | 99.14 | 1.1 | 1 | 4.984045 | SAMN52211686 |
| 1938688-1 | Lille | Lille | 4.998889 | 99.50 | 1.1 | 2 | 4.985821 | SAMN52211706 |
| 2002508-1 | Lille | Lille | 4.962091 | 99.60 | 1.1 | 3 | 4.945816 | SAMN52211728 |
| 1827333-1 | Hadar | Hadar | 4.71825 | 99.34 | 0.54 | 1 | 4.71825 | SAMN52211668 |
| 1830060-2 | Hadar | Hadar | 4.722889 | 99.09 | 0.54 | 1 | 4.722889 | SAMN52211669 |
| 1941725-1 | Hadar | Hadar | 4.719437 | 98.51 | 0.54 | 1 | 4.719437 | SAMN52211724 |
| 1941725-2 | Hadar | Hadar | 4.71149 | 98.65 | 0.6 | 1 | 4.71149 | SAMN52211725 |
| 1941725-3 | Hadar | Hadar | 4.719389 | 98.90 | 0.54 | 1 | 4.719389 | SAMN52211726 |
| 1835423-3 | Senftenberg | Senftenberg | 4.925674 | 99.27 | 1.55 | 4 | 4.891728 | SAMN52211671 |
| 1934052-2 | Senftenberg | Senftenberg | 4.918889 | 99.29 | 1.15 | 2 | 4.907854 | SAMN52211698 |
| 1940325-1 | Senftenberg | Senftenberg | 4.931334 | 99.29 | 1.26 | 3 | 4.860845 | SAMN52211721 |
| 1940325-2 | Senftenberg | Senftenberg | 4.865981 | 99.28 | 1.23 | 2 | 4.860766 | SAMN52211722 |
| 1940325-3 | Senftenberg | Senftenberg | 4.892248 | 99.28 | 1.2 | 3 | 4.860831 | SAMN52211723 |
| 1823843-2 | Agona | Agona | 4.9239 | 99.57 | 0.57 | 2 | 4.815474 | SAMN52211666 |
| 1939748-1 | Agona | Agona | 4.923892 | 99.66 | 0.54 | 2 | 4.815461 | SAMN52211719 |
| 1939748-3 | Agona | Agona | 4.923889 | 99.65 | 0.57 | 2 | 4.815455 | SAMN52211720 |
| 2037399-1 | Agona | Agona | 4.815596 | 95.85 | 0.71 | 1 | 4.815596 | SAMN52211737 |
| 2037399-4 | Agona | Agona | 4.815053 | 94.54 | 1.07 | 1 | 4.815053 | SAMN52211738 |
| 1938700-1 | Braenderup | Braenderup | 4.905336 | 98.14 | 0.26 | 5 | 4.825745 | SAMN52211711 |
| 1938700-2 | Braenderup | Braenderup | 4.846088 | 98.36 | 0.42 | 5 | 4.786777 | SAMN52211712 |
| 1938700-3 | Braenderup | Braenderup | 4.804697 | 98.44 | 0.42 | 5 | 4.788542 | SAMN52211713 |
| 1938700-4 | Braenderup | Braenderup | 4.80919 | 98.01 | 0.31 | 5 | 4.789305 | SAMN52211714 |
| 2037403-1 | Braenderup | Braenderup | 4.704694 | 98.04 | 1.06 | 1 | 4.704694 | SAMN52211739 |
| 1805577-5 | Rissen | Rissen | 4.992317 | 99.38 | 1.24 | 8 | 3.183764 | SAMN52211663 |
| 1826127-1 | Rissen | Rissen | 5.022446 | 99.26 | 2.76 | 8 | 4.838492 | SAMN52211667 |
| 1843855-5 | Rissen | Rissen | 4.875053 | 98.85 | 1.24 | 8 | 2.939529 | SAMN52211680 |
| 1921700-4 | Rissen | Rissen | 5.010676 | 99.31 | 1.24 | 7 | 3.194132 | SAMN52211685 |

| 1934345-3 | Rissen | Rissen | 4.998723 | 99.59 | 1.21 | 10 | 3.183806 | SAMN52211700 |
| --- | --- | --- | --- | --- | --- | --- | --- | --- |
| 1836504-1 | Ohio | Ohio | 4.754073 | 98.75 | 0.38 | 2 | 4.751774 | SAMN52211673 |
| 1839416-4 | Ohio | Ohio | 4.761645 | 98.36 | 0.41 | 2 | 4.759346 | SAMN52211675 |
| 1933359-3 | Ohio | Ohio | 4.754167 | 99.19 | 0.41 | 2 | 4.751802 | SAMN52211693 |
| 1934052-4 | Ohio | Ohio | 4.751702 | 98.19 | 0.38 | 1 | 4.751702 | SAMN52211699 |
| 1937074-1 | Ohio | Ohio | 4.754023 | 98.74 | 0.44 | 2 | 4.75172 | SAMN52211704 |
| 1934051-1 | Heidelberg | Heidelberg | 4.896562 | 99.59 | 0.44 | 5 | 4.751271 | SAMN52211694 |
| 1934051-2 | Heidelberg | Heidelberg | 4.809143 | 99.58 | 0.44 | 4 | 4.751296 | SAMN52211695 |
| 1934051-3 | Heidelberg | Heidelberg | 4.82184 | 99.57 | 0.44 | 4 | 4.751284 | SAMN52211696 |
| 1934051-4 | Heidelberg | Heidelberg | 4.809565 | 99.44 | 0.44 | 4 | 4.751273 | SAMN52211697 |
| 2002963-2 | Heidelberg | Heidelberg | 4.801426 | 99.18 | 0.41 | 3 | 4.756588 | SAMN52211729 |
| 1737637-4 | Enteritidis | Enteritidis | 4.793628 | 99.53 | 0.50 | 8 | 4.679295 | SAMN52211660 |
| 1830309-3 | Enteritidis | Enteritidis | 4.738773 | 99.09 | 0.53 | 2 | 4.679423 | SAMN52211670 |
| 1806503-4 | Enteritidis | Enteritidis | 4.738779 | 99.55 | 0.53 | 2 | 4.679425 | SAMN52211664 |
| 1927910-1 | Enteritidis | Enteritidis | 4.789114 | 99.16 | 0.53 | 3 | 4.712285 | SAMN52211689 |
| 1927913-3 | Enteritidis | Enteritidis | 4.771698 | 99.07 | 0.53 | 2 | 4.712342 | SAMN52211690 |

**Supplementary Table S1.** *Salmonella* serovars prediction using Oxford Nanopore Technology platform and corresponding genome assembly statistics.
